# Cuproptosis-related gene *SLC31A1* expression correlates with the prognosis and tumor immune microenvironment in glioma

**DOI:** 10.1007/s10142-023-01210-0

**Published:** 2023-08-23

**Authors:** Jun Wang, Shenglun Li, Yuduo Guo, Chao Zhao, Yujia Chen, Weihai Ning, Jingjing Yang, Hongwei Zhang

**Affiliations:** Department of Neurosurgery, Sanbo Brain Hospital, Capital Medical University, Beijing, China

**Keywords:** Glioma, Cuproptosis, Immune, Tumor microenvironment, *SLC31A1*, Proliferation, Migration, Apoptosis

## Abstract

Cuproptosis is a newly discovered form of cell death. It is regulated by a string of genes. The genes are identified to influence the tumor progression, but in glioma, the cuproptosis-related genes are little studied. The Cancer Genome Atlas (TCGA) and the Genotype-Tissue Expression (GTEx) were used to screen for *SLC31A1* gene expression in glioma and healthy tissue samples. The results were validated using the Gene Expression Omnibus (GEO) and quantitative real-time polymerase chain reaction (qPCR). The Human Protein Atlas (HPA) and the National Cancer Institute’s Clinical Proteomic Tumor Analysis Consortium (CPTAC) were used to validate our results at the protein level. Multivariable analysis and Kaplan–Meier survival curves were used to examine the relationship among *SLC31A1* gene expression, clinical parameters, and survival rates. The online Search Tool for the Retrieval of Interacting Genes/Proteins (STRING) was used to find the genes and proteins that correlate to *SLC31A1*. The immune infiltration analysis was performed using the Tumor Immune Estimation Resource (TIMER) databases. Small interfering RNA was used to knock down the SLC31A1 expression, and the cell proliferation, apoptosis, and migration were analyzed using cell counting kit-8, flow cytometry, and transwell. The glioma patients have higher *SLC31A1* expression levels, which increase as the World Health Organization (WHO) grade escalates. The survival analysis illustrates that the *SLC31A1* gene expression negatively correlates with overall survival (OS), progression-free survival (PFS), and disease-specific survival (DSS). The immune infiltration analysis shows the *SLC31A1* gene positively correlates with T helper 2 (Th2) cells, macrophages, and M2-type macrophages and negatively correlates with plasmacytoid dendritic cells (pDCs), natural killer (NK) CD56bright cells, and CD8 T cells. The in vitro KD experiment shows the *SLC31A1* knockdown depressed the glioma cell proliferation and migration and promoted the apoptosis rate. The *SLC31A1* gene expression can shorten the survival time of glioma patients. In vitro study shows that *SLC31A1* can promote cell proliferation, and migration, and depress the cell apoptosis of glioma cells. It also can promote the formation of a tumor-suppressive microenvironment.

**Supplementary Information:**

The online version contains supplementary material available at 10.1007/s10142-023-01210-0.

## Background

Cuproptosis is a recently discovered form of programmed cell death regulated by a set of genes known as cuproptosis-correlated genes (CRGs). While several studies have explored the role of CRGs in various cancers, the extent of research on the *SLC31A1* gene is limited. Glioma, a type of brain tumor, is known to be associated with dysregulated cell death pathways, making it a prime candidate for investigation of the role of *SLC31A1*.To this end, we analyzed data from online databases to investigate the relationship between *SLC31A1* gene expression and clinical outcomes and the tumor microenvironment in glioma patients. Our results revealed that high expression of the *SLC31A1* gene is associated with unfavorable outcomes and an immunosuppressive tumor microenvironment. These findings suggest that *SLC31A1* could be a valuable target for further investigation in the development of novel therapies for glioma.

## Introduction

Gliomas are the most common primary tumors in the brain, accounting for 81% of central nervous system (CNS) malignancies (Ostrom, et al. 2018). The current standard therapy interventions, including surgery, radiotherapy, and systemic chemotherapy, cannot convert the most treatment resistance of glioma (Jiang et al. 2021; Armstrong et al. 2020). One of the many reasons is the glioma immunosuppressive tumor microenvironment (TME) (Quail and Joyce 2017). As previously reported, the TME of glioma is immunosuppressive and is known as one of the cold immune tumors (D'Alessio, et al. 2019; Xiong et al. 2022). More key molecules need to be identified to understand the TME of glioma and improve the outcomes of glioma patients.

Copper is an essential element in cells (Lewinska-Preis et al. 2011). There is a balance in copper metabolism, and if this balance is broken, it may cause cell death, called cuproptosis. Furthermore, the scientists revealed that the cuproptosis is regulated by a series of genes, including *FDX1, LIPT1, LIAS, DLD, DBT, DLAT, GCSH, DLST, ATP7A, ATP7B SLC31A1* (solute carrier family 31 member 1), *PDHA1*, and *PDHB* (Tsvetkov et al. 2022). Most genes, such as FDX1, have been studied in different cancers to different degrees (Wang et al. 2022a; Zhang et al. 2022a; Zhang et al. 2022b; Song et al. 2022). Besides, we noticed that *SLC31A1* had been studied in different cancers and strongly correlated with poor prognosis (Li et al. 2022a; Kong et al. 2023). However, in glioma, the *SLC31A1* has not been studied in detail.

In this study, we used data from public datasets, including the Cancer Genome Atlas (TCGA), Gene Expression Omnibus (GEO), the Human Protein Atlas (HPA), the Chinese Glioma Genome Atlas (CGGA), and the Clinical Proteomic Tumor Analysis Consortium (CPTAC) to investigate the association between *SLC31A1* gene expression and clinical outcomes in glioma patients. Then, we tested the *SLC31A1* gene expression difference between glioma cell lines U87, LN229, U251, U343, and the average human fetal glial cell line SVGp12 using a Real-time Quantitative PCR Detecting System. We then used data from the Tumor Immune Estimation Resource (TIMER) and the Gene Expression Profiling Interactive Analysis (GEPIA) databases to analyze the relationship between *SLC31A1* gene expression and immune cell infiltration and the associated gene marker sets. Furthermore, we analyzed the *SLC31A1*-interacting protein network using the STRING (Search Tool for the Retrieval of Interacting Genes/Proteins) platform. A high *SLC31A1* gene level is related to infiltrating immune cell changes in glioma tissues, indicating a dismal prognosis. Thus, it is plausible that the *SLC31A1* gene defect may debilitate antitumor immune effects for glioma. *SLC31A1*-related targeting may be a viable treatment approach in glioma in combination with immunotherapy.

## Results

### SLC31A1 gene expression was increased in glioma as opposed to normal samples

**Fig. 1:**
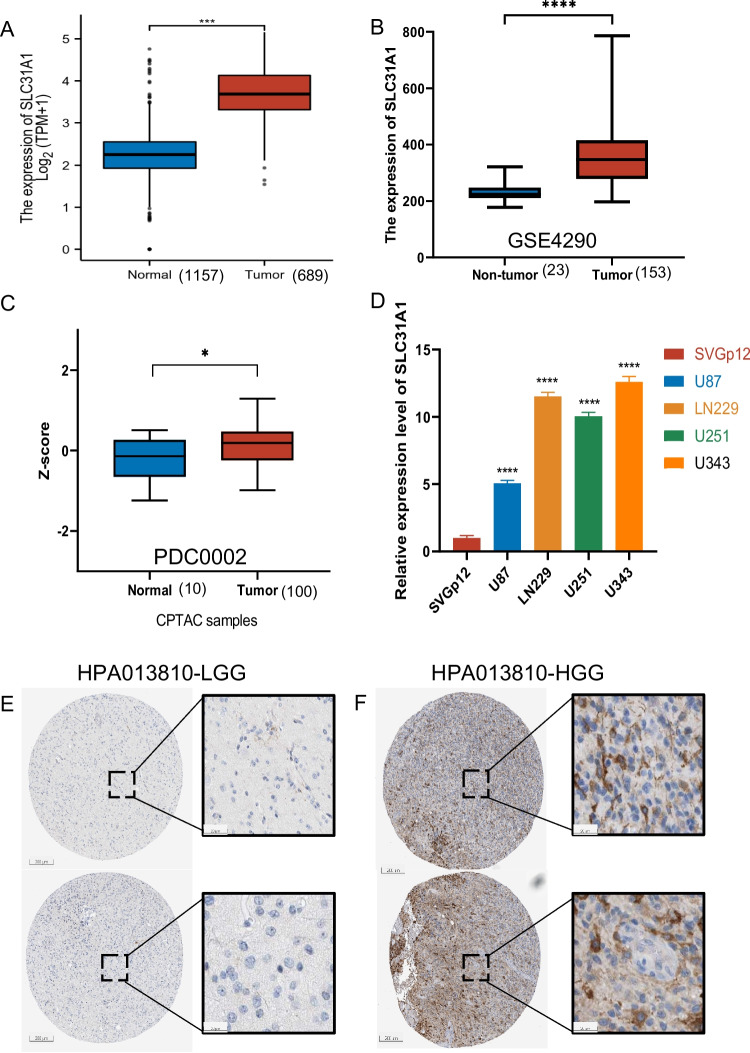
SLC31A1 is upregulated in glioma. The expression difference between normal tissue and cancer in mRNA (**A**, **B**, and **D**) and protein (**C**) level. The expression difference between LGG and HGG illustrated by IHC image (*p < 0.05, **p < 0.01, ***p < 0.001, and ns, no statistical difference)

To determine whether *SLC31A1* gene expression is higher in glioma patients, we analyzed data from different datasets and levels; at first we analyzed the *SLC31A1* gene expression in glioma samples using the data from the TCGA dataset and compared it to the normal tissue samples from the GTEx dataset (Fig. 1A) (*N* = 1157, *T* = 689). The GEO datasets were used to test the accuracy of the tendency (Fig. 1B) (*N* = 23, *T* = 153). As demonstrated, the *SLC31A1* gene expression at the mRNA level is upmodulated in glioma patients (*p* < 0.001) (Fig. 1A and B). It has been shown that the *SLC31A1* gene may play a key role in glioma. Then, we further tested the *SLC31A1* protein expression level by using data from the CPTAC database (*N* = 10, *T* = 100), and we found that the *SLC31A1* protein is upmodulated in glioma samples (*p* = 0.0205) (Fig. 1C). These results support that the *SLC31A1* is a glioma-promotive gene, as the previous study has partially put it (Li, et al. 2023). Cell lines were also used to confirm the difference in expression between glioma and normal control cell lines. We found that the *SLC31A1* gene expression is significantly upregulated (*p* < 0.0001) in glioma cell lines (Fig. 1D). Additionally, to further determine the expression level of *SLC31A1* at the protein level, we used the histochemical data from HPA. As is shown in Fig. 1E and 1F, the positive rates of *SLC31A1* are higher in HGG (high-grade glioma) patients than in LGG (low-grade glioma) patients. All of the results indicate that the *SLC31A1* gene is associated with glioma (Table 1).

**Table 1:** Proteins interacting with SLC31A1 in glioma tissue. Annotation of proteins that interact with SLC31A1, along with their respective co-expression scores

Gene	Annotation	Score
ATOX1	Copper transport protein ATOX1	0.938
ATP7B	Copper-transporting ATPase 2	0.887
ATP7A	Copper-transporting ATPase 1	0.887
COX17	Cytochrome c oxidase copper chaperone	0.827
CCS	Copper chaperone for superoxide dismutase	0.768
SLC11A2	Natural resistance-associated macrophage protein 2	0.738
MTF1	Metal regulatory transcription factor 1	0.703
SLC22A2	Solute carrier family 22 members 2	0.687
ZBED3	Zinc finger BED domain-containing protein 3	0.676
CP	Ceruloplasmin	0.675

### Association between SLC31A1 expression and clinical parameters

**Fig. 2:**
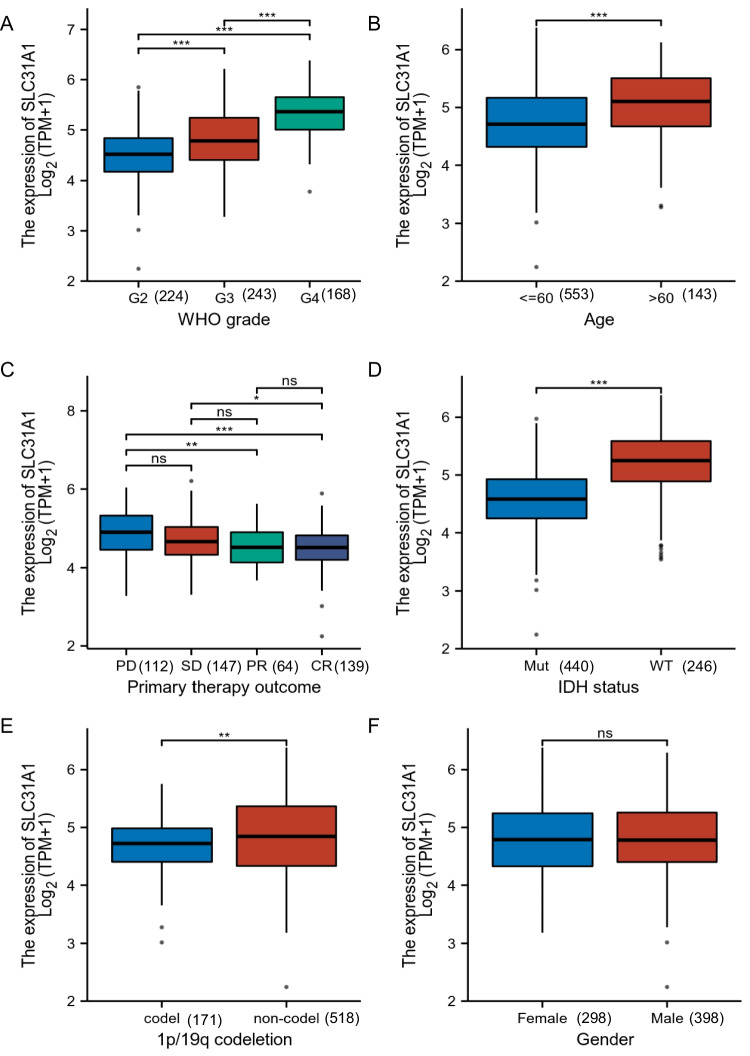
Association between SLC31A1 expression and clinical-pathological parameters of glioma. The association of SLC31A1 and different clinical parameters such as WHO grade (**A**), age (**B**), primary therapy outcome (**C**), IDH status (**D**), 1p/19q codeletion (**E**), and gender (**F**)

Based on the previous data, it has been found that *SLC31A1* is upmodulated in glioma patients. Therefore, we investigated the difference in *SLC31A1* gene expression in patients with different clinical parameters, based on the expression differences of *SLC31A1*, we separated the patients into low- and high-expression groups. The clinicopathological characteristics in patients with glioma are shown in Table 2. We used the Kruskal–Wallis and Wilcoxon signed-rank tests to determine the relationship between *SLC31A1* expression and clinical parameters. We found that higher WHO grade (G2 = 224, G3 = 243, G3 = 168), higher age (≤ 60 = 553, > 60 = 143), and progressive disease (PD) (PD = 112, SD = 147, PR = 64, CR = 139) were associated with high-level *SLC31A1* expression (Fig. 2A, B, and C). The SLC31A expression level escalates as the WHO grade increases. The results indicate the *SLC31A1* gene is correlated with bad outcomes in glioma patients. Also, the *SLC31A1* expression level is higher in patients with *IDH* WT status (WT = 246, Mut = 440) and 1p/19q non-codeletion (codel = 171, non-codel = 518), which were proven to have a strong correlation with unfavorable prognostic outcomes in glioma patients (*p* < 0.01) (Fig. 2D and E). The data from CGGA confirmed the same tendency (Figure S1). Nevertheless, there is no significant *SLC31A1* difference between male (398) and female (298) patients (Fig. 2F). Overall, our analysis demonstrated that the *SLC31A1* gene expression positively correlates with WHO grades, patients’ age, and bad primary therapy outcomes, which are indicators of poor outcomes. These results confirm *SLC31A1* gene has a tremendous prognostic value in glioma patients.

**Table 2:** Association of SLC31A1 expression with clinicopathological characteristics in patients with glioma

Characteristic	Low expression of SLC31A1	High expression of SLC31A1	*p*
*n*	348	348	
WHO grade, *n* (%)			< 0.001
G2	158 (24.9%)	66 (10.4%)	
G3	123 (19.4%)	120 (18.9%)	
G4	22 (3.5%)	146 (23%)	
IDH status, *n* (%)			< 0.001
WT	54 (7.9%)	192 (28%)	
Mut	292 (42.6%)	148 (21.6%)	
1p/19q codeletion, *n* (%)			0.018
Codel	100 (14.5%)	71 (10.3%)	
Non-codel	247 (35.8%)	271 (39.3%)	
Primary therapy outcome, *n* (%)			< 0.001
PD	51 (11%)	61 (13.2%)	
SD	87 (18.8%)	60 (13%)	
PR	44 (9.5%)	20 (4.3%)	
CR	102 (22.1%)	37 (8%)	
Gender, *n* (%)			0.818
Female	147 (21.1%)	151 (21.7%)	
Male	201 (28.9%)	197 (28.3%)	
Age, *n* (%)			< 0.001
≤ 60	304 (43.7%)	249 (35.8%)	
> 60	44 (6.3%)	99 (14.2%)	
Age, median (IQR)	41 (33, 52)	52.5 (37, 62)	< 0.001

### Prognostic relevance of SLC31A1 expression in glioma

**Fig. 3:**
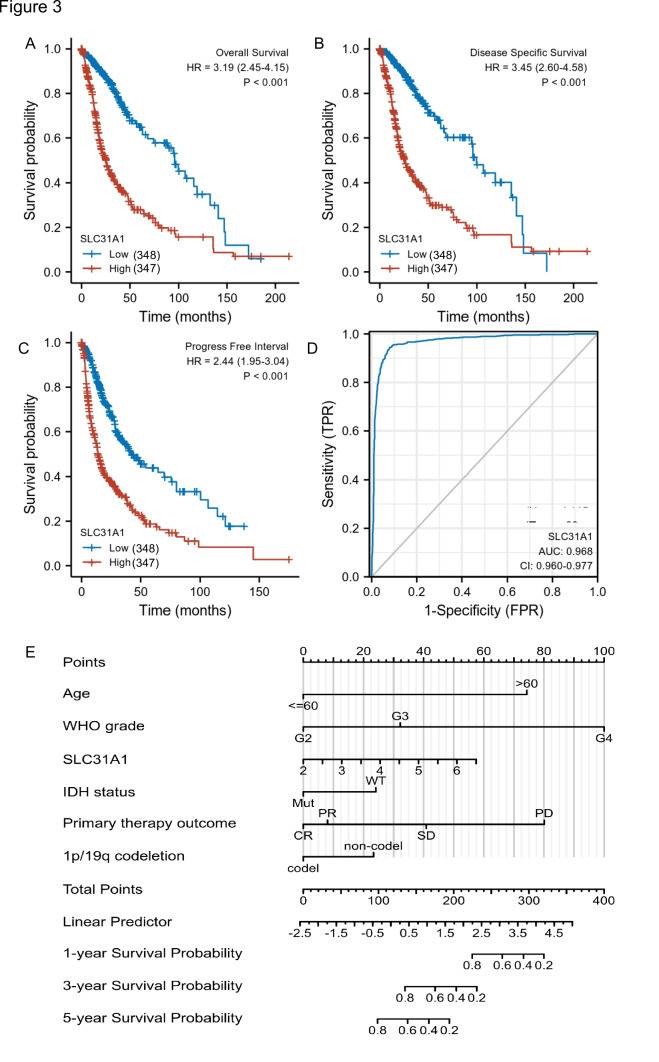
The patients with higher SLC31A1 expression is more likely to have a worse prognosis. Patients with high SLC31A1 expression had unfavorable prognosis indicators than patients with low SLC31A1 expression, including shorter overall survival (OS) (**A**), progression-free interval (DSS) (**B**), and disease-specific survival (PFS) (**C**) (Log-rank *p* < 0.001). Receiver operating characteristic curve for SLC31A1 expression in normal samples (obtained using GTEx data) and adjoining glioma tissues and samples (**D**). A multivariate analysis nomogram based on clinical features associated with SLC31A1 expression (**E**)

The former results show that the *SLC31A1* gene is associated with the prognostic parameters; we then further investigated the prognostic survival value of *SLC31A1* in glioma patients. We ranked the TCGA glioma patient samples by the expression level of *SLC31A1*, and the glioma cohort was classified into low- and high-expression groups according to the expression levels of *SLC31A1*. Then, we compared the prognosis difference between the two groups. As the pictures show, the survival curves of the low *SLC31A1* group are above the high *SLC31A1* group in OS, DSS, and PFS, which means the low *SLC31A1* group survives longer than the high *SLC31A1* group (Fig. 3A, B, C) (low = 348, high = 347), the data from CGGA shows the same tendency (Figure S2). The results mean that the high-level expression of *SLC31A1* was associated with unfavorable OS (hazard ratio (HR) = 3.19(2.45–4.15), *p* < 0.001, Fig. 3A), DSS (HR = 3.45 (2.60–4.58), *p* < 0.001, Fig. 3B), and PFS (HR = 2.44 (1.95–3.04), *p* < 0.001, Fig. 3C). Then, a receiver operating characteristic (ROC) curve was constructed to examine the diagnostic significance of *SLC31A1* expression by comparing *SLC31A1* expression in normal tissue specimens (data obtained from GTEx) and glioma tissues (from the TCGA database). As is demonstrated that the area under the curve (AUC) value for *SLC31A1* levels was 0.968 (confidence interval = 0.960–0.977), which means strong potential for diagnostic use (Fig. 3D) (*N* = 1157, *T* = 689). Following that, a clinical prognostic risk score for glioma was created using age, WHO grade, *IDH* status, 1p/19q codeletion, primary therapy outcome, and *SLC31A1* expression. We found that the *SLC31A1* has great prognostic predicted efficiency in glioma patients (Fig. 3E). Similarly, we also draw the same conclusion in univariate and multivariate analyses (using the Cox regression model) (Figure S3). In this part, we found that the *SLC31A1* is an independent risk factor of glioma patients, which is useful for the outcome prediction of glioma. But how this gene acts is still unclear; we then further investigated the functions of *SLC31A1*.

### The functions of SLC31A1

**Fig. 4:**
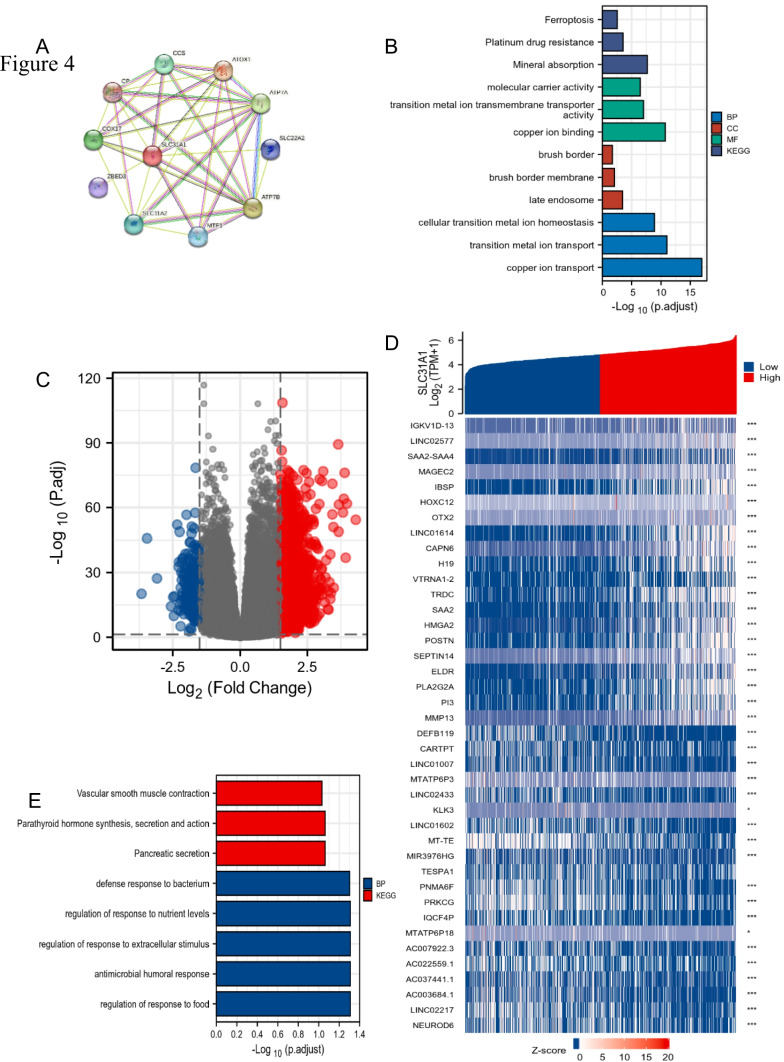
The functions of SLC31A1. The SLC31A1-correlated genes from STRING database (**A**). The GO/KEGG analyses of the SLC31A1-correlated genes (**B**). The gene expression difference correlates to SLC31A1 in glioma (**C**). The top 40 genes correlate to SLC31A1 (**D**). The GO and KEGG analyses using the top correlating genes

To better understand the functions of the *SLC31A1* gene in glioma, we analyzed the genes related to the *SLC31A1* by the STRING network. As is illustrated in Fig. 4A, the most top 10 genes related to *SLC31A1* are *ATOX1, ATP7B, ATP7A, COX17, CCS, SLC11A2, MTF1, SLC22A2, ZBED3*, and *CP*. Annotations and their co-expression scores are displayed in Table 1. We then used the GO-KEGG enrichment analysis to analyze the functions of these genes. We found that the genes are mainly enriched to copper ion transportation pathways (Fig. 4B), showing that the *SLC31A1* is a key gene for Cu^2+^ concentration regulation, which can be supported by a former study (ZHOU, B. and J. GITSCHIER 1997). To better understand the biological role of the *SLC31A1* gene in glioma, we analyzed the whole-genome profile of glioma patients from TCGA. It was discovered that 285 genes are downregulated, and 1686 genes are upregulated, linked to the *SLC31A1* gene expression. The gene expression heat map displayed the top 40 aberrant gene expression levels (logFC > 1.5 and padj < 0.05) (Fig. 4D). To further investigate the possible functions of *SLC31A1* in glioma, the enrichment analysis was performed based on the *SLC31A1* gene expression results. The BP primarily associated with the *SLC31A1* gene was the regulation of response to food, antimicrobial humoral response, regulation of response to extracellular stimulus, regulation of response to nutrient levels, and defense response to the bacterium, among others (Fig. 4E). The results show a strong correlation to immune response, which is a strong influence factor of glioma progression; then, we further investigated the correlation between *SLC31A1* and the TME of glioma.

### The association between the SLC31A1 gene expression and immune cell infiltration

**Fig. 5:**
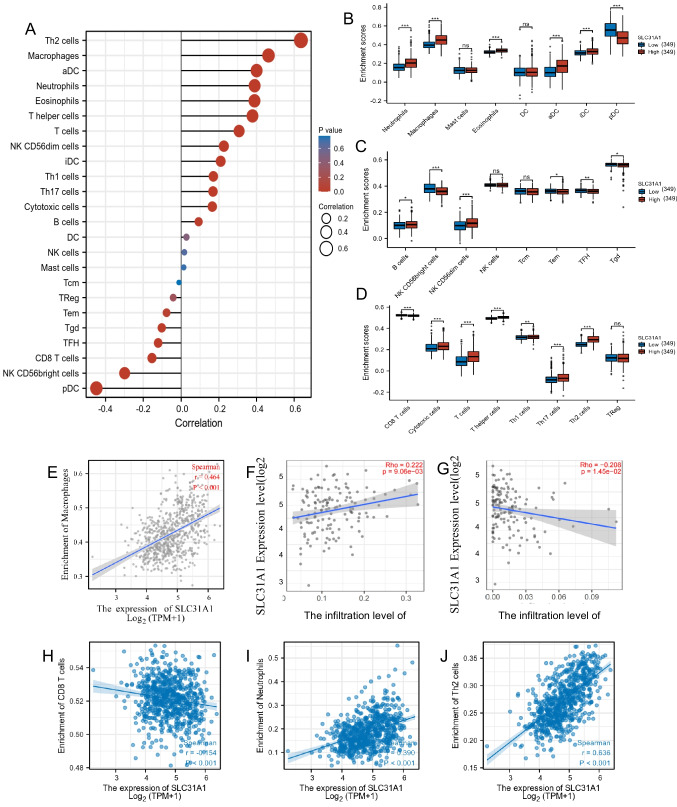
SLC31A1 has a strong correlation with glioma TME. The correlation between SLC31A1 gene and glioma immune infiltration status (**A**). The infiltration difference of immune cells between the low and high SLC31A1 expression groups (**B**–**D**). Relationships between SLC31A1 gene expression and immune cells

As previously described, the genes associated with *SLC31A1* analyzed have shown a strong correlation with immunity (Li et al. 2022b). We then explored the relationship between *SLC31A1* gene expression and 24 distinct immune subtypes in glioma using the online analysis tool of the TIMER2.0 database. We found that the *SLC31A1* gene expression had a positive correlation with Th2 cells, macrophages, aDC cell infiltration, and a strong inverse correlation with pDC, NK CD56bright cells, and cytotoxic CD8 + T cell infiltration, among other cells (Fig. 5A, B–D). In the TME of glioma, the tumor-associated macrophages (TAMs) are critical; their volume may take up 30% of the glioma tissue (Hambardzumyan et al. 2016); the macrophages can be divided into M0, M1, and M2 types by the biomarkers and functions they expressed. Previous studies demonstrated that the M1-type macrophage is immune-promotive (Wang et al. 2022b) and the M2-type macrophage is immune-suppressive (Yunna et al. 2020). Th2 cells are also important tumor-promoting cells that can promote the M2 polarization of macrophage by secreting IL4 and IL13 cytokines (Gordon and Martinez 2010; Stark et al. 2019). As we investigated, the Th2 cell infiltration positively correlates to *SLC31A1* gene expression. The M2-type macrophages are also upregulated as the *SLC31A1* gene expression increases, indicating that the *SLC31A1* may promote macrophage polarization by promoting the IL4 and IL13 secretion of Th2 cells. Further investigation illustrated that the *SLC31A1* gene expression level differs in immune cell infiltration; the most correlated are neutrophils, eosinophils, T cells, and T helper cells, among others (Fig. 5B–D). To investigate the possible function of the *SLC31A1* gene in influencing the infiltration status of different immune cells in glioma, we analyzed data from the TIMER and GEPIA databases to uncover the link between the *SLC31A1* gene and different immune cell infiltration, including DCs, NK cells, M1/M2 macrophages, T cells (general), neutrophils, tumor-associated macrophages (TAMs), B cells, monocytes, and CD8 + T cells, in glioma. Besides, this study also evaluated different T cell subtypes, such as exhausted T cells, Tregs, Th1, Th2, Th9, Th17, Th22, and TFh. According to our results, the expression of most immune cell markers for various types of M1/M2 macrophages, TAMs, DCs, and T cells is linked to the *SLC31A1* gene expression level in glioma. Among them, the Th2 and macrophage cells are the most relevant cells, which shows that the *SLC31A1* gene may have a strong influence on the TME of glioma.

### SLC31A1 knocks down depressed glioma proliferation and migration, promoted the glioma apoptosis

**Fig. 6:**
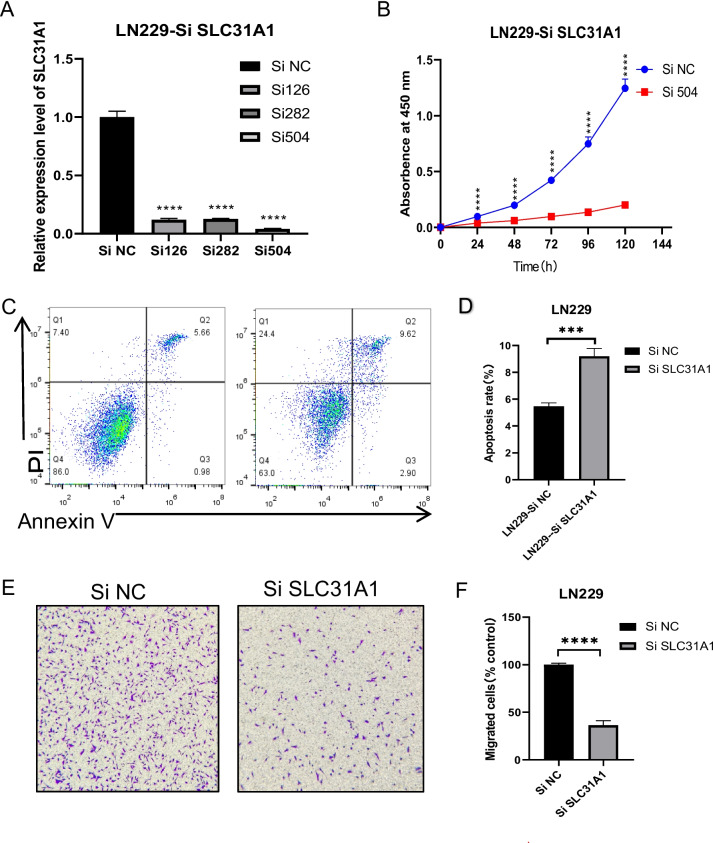
SLC31A1 knockdown depressed glioma viability and migration. We knocked down (KD) the SLC31A1 gene expression in glioma cell line LN229 by siRNA and the KD efficiency was detected by the qPCR method (**A**). The cell proliferation difference was detected by the CCK-8 Kit (**B**). The SLC31A1 KD promoted glioma cell apoptosis (**C**, **D**). The cell migration rate was analyzed by transwell (**E**, **F**). All the experiments have been repeated for three times

As SLC31A1 is crucial in glioma, we then tested the *SLC31A1* gene function by in vitro experiments using LN229 and U251 cell lines. We knocked down (KD) the *SLC31A1* in LN229 and U251 by small interferon RNA (siRNA), the Si504 shows strong knockdown efficiency than the other two siRNAs in both LN229 (95.7%) and U251 (95.3%) cell lines (Fig. 6A and Figure S4 A). Then, we analyzed the cell proliferation, apoptosis, and migration after knocking down the *SLC31A1*. Compared to the NC group, the si504 group shows a slower growth speed in both LN229 and U251 (Fig. 6B and Figure S4 B), and the flow cytometry assay shows the si *SLC31A1* group has more apoptotic cell rates than the NC group (Fig. 6B and C, Figure S4 B & C). The transwell assay also shows the si504 group has less penetrated cells (Fig. 6E and F, Figure S4 E & F). The results show that the proliferation and migration were both depressed in LN229 and U251. KD *SLC31A1* also promoted glioma cell apoptosis. This means the *SLC31A1* may function by promoting the proliferation and migration of glioma cells. And the *SLC31A1* gene might be important for the resistance of apoptosis in glioma. The experiments were repeated for 3 times and the data were processed by t test using Prism. A *p* value < 0.05 was considered statistically significant (*p < 0.05; **p < 0.01; ***p < 0.001; and ****p < 0.0001).

## Discussion

Glioma is a refractory disease. Currently, our cognition of glioma is still limited. Scientists are trying to find new ways to understand and treat this deathful disease. Many factors are considered to be very important in glioma progression, such as gene mutation (Eckel-Passow et al. 2015), metabolism (Bi et al. 2020; Yu et al. 2023), tumor microenvironment (Friebel et al. 2020), and many kinds of cell death (Liu et al. 2022; Li et al. 2023; Abulaiti et al. 2023). Cuproptosis is a novel mode of cell death, especially in tumor cells; recent studies show that cuproptosis occurs in tumors and strongly impacts patients’ outcomes. Further research has revealed that cuproptosis can promote immune-suppressive microenvironment formation in different cancers (Du et al. 2022; Lv et al. 2022; Wang et al. 2022c). But only a few studies have examined its effects on glioma. The *SLC31A1* encodes a membrane protein that can transport copper ions into the cells (ZHOU, B. and J. GITSCHIER 1997). It promotes copper ion metabolism in glioma cells; as the former research shows, the *SLC31A1* gene is an essential gene for cuproptosis. We hypothesize that the *SLC31A1* gene can promote the progression of glioma. As we analyzed, the high-*SLC31A1* group glioma patients had worse outcomes than those with lower *SLC31A1* gene expression levels. This finding might indicate that the glioma cell can resist cuproptosis by transporting the Cu^2+^ into the cells, which may be the molecular function of the *SLC31A1* that contributes to glioma progression.

The previous study showed that the *IDH* mutation and 1p/19q codeletion significantly correlate with better outcomes in glioma patients (Sasmita et al. 2018; Jenkins et al. 2006). So we analyzed the relationship between *SLC31A1* expression level and *IDH* mutation, 1p/19q codeletion. We found that the *SLC31A1* expression level is lower in the *IDH* mutation group and 1p/19q codeletion group, which means that the *SLC31A1* might influence the outcomes of glioma patients by influencing *IDH* mutation and 1p/19q codeletion.

In our research, we also explored the relationship between *SLC31A1* gene expression and the outcomes of glioma patients. The high *SLC31A1* expression level strongly correlates with bad outcomes in glioma patients. Following that, we further investigated the possible mechanism of how the *SLC31A1* gene expression influences glioma progression. The in vitro knockdown experiment also shows that the *SLC31A1* KD strongly impaired the proliferation and migration of glioma cells, which is the same as we analyzed by the in silico data. This means the *SLC31A1* may promote glioma progression by enhancing the proliferation and migration of glioma cells. After that, we sorted the top 40 genes that mostly correlate with the *SLC31A1* gene in glioma. Then, we did a gene enrichment analysis by using these genes. The result indicates that these genes have a strong correlation with immune response. That means the *SLC31A1* gene might influence the glioma progression by reshaping the immune microenvironment. As former researches show (Quail and Joyce 2017; Yu et al. 2023; Klemm, et al. 2020), the TME of glioma plays a significant role in glioma progression. The former research has illustrated that the TME of glioma is suppressive, and the suppressive TME can promote the immune escape of glioma cells besides, as the brain is an immune-privileged organ. The TME of glioma is complicated, there are lots of investigations about it, and scientists have found many regulating factors of the glioma TME. As we investigated, the cuproptosis-correlated gene *SLC31A1* is related to the immune response, but whether and how it acts in the TME of glioma is not clearly investigated. So, we then investigated the correlation between immune cell infiltration and SLC31A gene expression. We found that the *SLC31A1* gene influences many kinds of immune cell infiltration. Mostly the tumor-associated macrophages (TAMs), T helper 2 (Th2) cells, plasmacytoid dendritic cells (pDCs), NK CD56bright cells, and CD8 T cells. Tumor-associated macrophage (TAM) refers to the macrophages recruited in the tumors; it also includes the three types of macrophages mentioned. Microglia and macrophages are the main component of glioma tumor–associated macrophages (TAMs) and have been categorized as M1 polarized (“classically activated”), M2 polarized (“alternatively activated”), and nonpolarized M0 macrophages (Ochocka et al. 2021). Among them, M0 macrophages are defined as undifferentiated macrophages with the potential to polarize into specific macrophage subtypes; M1 macrophages are classically activated, typically by IFN-γ or lipopolysaccharide (LPS), and produce proinflammatory cytokines, phagocytize microbes, and initiate an immune response (Mills, et al. 1950); M2 macrophages mainly secrete arginase-I, IL-10, and TGF-β and other anti-inflammatory cytokines, which have the function of reducing inflammation and contributing to tumor growth and immunosuppressive function (Hambardzumyan et al. 2016; Mosser and Edwards 2008; Roszer 2015). As we analyzed, in GBM, the *SLC31A1* gene expression positively correlates with M2 macrophage infiltration but negatively correlates with M1 macrophage infiltration, which may cause immune-suppressive TME formation and promote the immune escape of the glioma cells. Th cells can also influence the TME of glioma; they are divided into different subtypes by the cytokines they secrete, mostly the Th1 and Th2 cells (Zhu and Zhu 2020). As previous research described, the Th2 bias, which means the Th2 ratio increasing, is important for glioma progress. The Th2 cell infiltration level increases as the WHO grades increase (Shimato 2012). The Th2 cells can secret IL4 and IL13 cytokines with strong M2 polarization functions of macrophages (Walker and McKenzie 2018). Another study found that Th2 bias in glioma is associated with increased expression of STAT6, a transcription factor that regulates Th2 differentiation (Shimato 2012). In short, in glioma, the M2-type macrophages are proven to have a strong immune suppressive function (Vidyarthi et al. 2019; Xu et al. 2021). As we investigated, the *SLC31A1* gene expression positively correlates with Th2 cells, which indicated that the *SLC31A1* has a strong impact on the TME of glioma. However, the study has limitations. Firstly, the function of the *SLC31A1* gene in the glioma microenvironment is still not clear. Further investigations need to be done to understand the *SLC31A1* gene. Secondly, how the *SLC31A1* acts in glioma are still unclear, the analysis based on online data shows that the *SLC31A1* influenced the progression of glioma, but whether and how it regulates the cuproptosis and other cell physiological activities in glioma is not validated, the further investigation is needed.

## Conclusions

Our results indicate that the *SLC31A1* gene expression can promote the formation of an immune-suppressive glioma microenvironment. The results also show that the *SLC31A1* might be a critical molecular for the immune regulation of glioma. The medications targeting *SLC31A1* may be helpful for the treatment of glioma. Fully understanding the *SLC31A1* may be beneficial for clinical staging and personalized therapy.

## Materials and methods

### Cell lines

The human glioma cell lines U87, LN229, U251, and U343 and the normal human fetal glial cell line SVGp12 were purchased from ATCC, and all cell lines were cultured in Dulbecco’s Modified Eagle Medium (DMEM, Gibco) with 10% FBS (PAN, Germany) in a humidified chamber at 5% CO_2_ and 37 ℃.

## Data sources

### The TCGA database

The TCGA (https://portal.gdc.cancer.gov) is a public data platform for cancer genome projects. It provides clinical and pathological data on 33 types of cancer that are easy to obtain. We searched the TCGA database for clinical data and high-throughput RNA sequencing (RNA-seq) information on patients with glioma. Moreover, we used fragments per kilobase per million fragments mapped (FPKM) included in HTSeq to determine *SLC31A1* transcript expression levels. Furthermore, to further investigate the RNA-Seq gene expression level 3 HTSeqFPKM data of 703 patients with glioma, the clinical data were transformed into transcripts per million (TPM) reads. The database is public; therefore, the local ethics committee has no permission.

### The CGGA database

The CGGA (https://www.cgga.org.cn/) is a public data platform that has collected more than 2000 Chinese glioma patients’ clinical and gene expression data. We used the data from CGGA to test the conclusions drawn from the TCGA database.

### The GEO database

The GEO database (https://www.ncbi.nlm.nih.gov/geo/) is a public functional genomics data repository that supports MIAME-compliant data submissions. It is a comprehensive gene expression bank at the National Center for Biotechnology Information (NCBI). It provides gene expression data and clinical data of different patients, including glioma patients and healthy individuals. We used the data from the GEO database to examine the differences in *SLC31A1* expression levels between glioma and normal tissues.

### The CPTAC database

The CPTAC database (https:// proteomics.cancer.gov/programs/cptac/) is proteogenomic, which can provide proteome expression data of many kinds of tumors and normal tissues, including glioma and normal brain tissue. We downloaded the *SLC31A1* protein expression data and analyzed the expression difference between normal brain tissue and glioma.

### The HPA database

The HPA database (https://www.proteinatlas.org/) is a giant online database that contains extensive transcriptome and proteome information of different human specimens, including tissue, cell, and pathology samples. The HPA database can provide data on the cell-specific positions of the 44 normal tissues and the twenty most susceptible cancers. Moreover, the database provides information on protein immunohistochemistry in tumors and standard human tissue samples.

### Statistical analysis of clinical prognosis, model development, and assessment

We analyzed the prognostic parameters, such as OS, disease-specific survival (DSS), and progression-free interval (PFS), using the data of glioma patients from the TCGA in the Xiantao platform (https://www.xiantao.love/). These analyses were performed using the Cox regression and Kaplan–Meier methods. The low and high *SLC31A1* gene expression groups were separated by the median value. We used the Wilcoxon signed-rank sum test and logistic regression to analyze the relationship between clinical-pathological characteristics and *SLC31A1* expression. We used a multivariate Cox regression model to investigate the effect of *SLC31A1* gene expression on the likelihood of survival and other clinical variables. A *p* value less than 0.05 was set as the threshold for significance. The Cox regression model findings were combined with the independent prognostic variables obtained from the multivariate analysis, and the survival probabilities for 1, 3, and 5 years were projected using these data. The projected odds were compared to actual occurrences using calibration curves. The 45-degree line represented the most accurately predicted value.

### Protein interaction analysis in silico

The STRING platform (https://string-db.org/) was used for protein correlation data analysis. It provides integrated and consolidated PPI (protein–protein interaction) data. After importing the *SLC31A1* expression data into the STRING platform, we received information from the PPI network. The threshold was set at a confidence score greater than 0.7. To get a more accurate result of the *SLC31A1*-related genes, the *p* value was adjusted by the Benjamini–Hochberg method, only the genes with adjusted *p* value (padj) < 0.05 was considered significant.

### Analysis of the immune cell infiltration in silico

The research of Bindea G et al. showed that different cell markers could characterize different types of immune cells (Bindea et al. 2013). We then investigated the tumor infiltrated 24 different types of immune cells using the ssGSEA method using the data from TCGA. The Spearman correlation algorithm was used to compare immune cell infiltration levels between high and low *SLC31A1* gene expression. The link between *SLC31A1* gene expression level and immune infiltration, the association between infiltrating levels of immune cells, and the values obtained in various *SLC31A1* gene expression subgroups were analyzed in the module of the “Xiantao tool” based on the findings of immune infiltration, Xiantao tool Spearman correlation, and Wilcoxon signed-rank sum. A *p* value < 0.05 was considered statistically significant (*p < 0.05; **p < 0.01; ***p < 0.001; and ****p < 0.0001).

### Gene correlation analysis

GEPIA (http://gepia.cancer-pku.cn/index.html) is a web platform providing information on 9736 cancer types and 8587 specimens derived from TCGA and GTEx. It usually focuses on the analysis of the RNA-seq findings. The Gene and Isoform classes each specify the types of the corresponding number of types of genes and isoforms, which total 60,498 and 198,619, respectively. An investigation was conducted in the GEPIA database to confirm the relationship between the expression of the *SLC31A1* gene and various immune cell markers. The expression degree of the *SLC31A1* gene is shown along the x-axis, whereas the expression of other relevant genes is shown on the y-axis. Furthermore, using data from TIMER (http://cistrome.org/TIMER/), we confirmed the expression of genes with a strong relationship to *SLC31A1* gene expression in GEPIA. A *p* value < 0.05 was statistically significant (*p < 0.05; **p < 0.01; ***p < 0.001; and ****p < 0.0001).

### Quantitative real-time polymerase chain reaction (qPCR)

The total mRNAs were extracted with TRIzol reagent (Ambion, REF:15,596,018); then, we reversed the mRNAs to cDNAs using the cDNA Synthesis SuperMix kit (cat. E047-01B). We used 2 × RealStar Green Fast Mixture qPCR kit (GenStar, Cat: A301-101) to analyze the expression of the *SLC31A1* gene in different glioma cell lines, in which *GAPDH* was used as the housekeeping gene, and its reliability was determined by the computational program RefFinder (Xie et al. 2023). The primers are as follows:

#### *SLC31A1*

Forward: 5′-ATGGAACCATCCTTATGGAGACA-3′

Backward: 5′-GGAAGTAGCTTATGACCACCTGG-3′

#### *GAPDH*

Forward: 5′-TGACTTCAACAGCGACACCCA-3′

Backward: 5′-CACCCTGTTGCTGTAGCCAAA-3′

The thermal cycle condition for qPCR: 95 ℃, 10 min; 95 ℃, 15 s; 60 ℃, 60 s, 40cycles.

The experiment has been repeated three times.

### Knockdown of SLC31A1 in glioma cell lines

The siRNA targeting *SLC31A1* was ordered from the OBiO company. The sequences of siRNAs are shown behind. The glioma cell lines LN229 and U251 were evenly planted in 6-well plates (1 × 10^5^/well) in Dulbecco’s Modified Eagle’s Medium (Gibco) supplemented with 10% fetal bovine serum (PAN, Germany) and 100 μL/mL penicillin and streptomycin and placed at 37 °C with 5% CO2. siRNA of 110 pmol was transfected into per well; the transfection buffer was purchased from PolyPlus (jetPRIME® transfection reagent). The transfection was performed as suggested in the user guidance. The transfect efficiency was examined by the qPCR method. The experiment has been repeated for three times.

### SiRNA for human SLC31A1

#### Si126

Forward: 5′-GAUGCCUAUGACCUUCUACUUTT-3′

Backward: 5′-AAGUAGAAGGUCAUAGGCAUCTT-3′

#### Si282

Forward: 5′-GCGUAAGUCACAAGUCAGCAUTT-3′

Backward: 5′-AUGCUGACUUGUGACUUACGCTT-3′

#### Si504

Froward: 5′- CGGUACAGGAUACUUCCUCUUTT-3′

Backward: 5′-AAGAGGAAGUAUCCUGUACCGTT-3′

#### SiNC

Forward: 5′-UUCUCCGAACGUGUCACGUTT-3′

Backward: 5′-ACGUGACACGUUCGGAGAATT-3′

### CCK-8 assay

One thousand cells were seeded in 96-well cell plates and added CCK-8 solution (Beyotime biotechnology: C0010) at 24, 48, 72, 96, and 120 h. Two hours later, the OD value at 450 nm was measured by the microplate reader (Thermo-Fisher Scientific). The CCK-8 assay has been repeated for three times.

### Transwell assay

Cells in the logarithmic growth phase were adjusted to 2 × 10^4^ cells/well of 200 uL medium (without serum) and plated into the upper chamber (Corning: 3422). The lower chamber was added with 750 μL of medium (with 10% FBS), and then incubate the chamber at 37 °C for 20 h. Then, the migrated cells were fixed by 4% paraformaldehyde (LABLEAD: P4500, 20 min) and visualized by the 0.1% crystal violet (Solarbio: G1063, 20 min). The cells not penetrated were gently wept off by cotton. After drying, cells in four random visual fields were counted under an inverted microscope (Leica, Wetzlar, Germany) to obtain the mean value of cells. For the accuracy of our results, the transwell assay has been repeated for three times.

### Flow cytometry

Transfected cells were gently collected and adjusted to a cell concentration of 1 × 10^5^ cells/195 uL conjugation buffer (C1062M, Beyotime). Cells were stained with 5 uL Annexin V-fluorescein isothiocyanate (FITC) and 10 uL propidium iodide (PI) at RT (room temperature) for 15 to 20 min. The FITC and PI both positive cells were considered apoptotic and detected using a flow cytometer (CytoFLEX, Beckman Coulter). The apoptosis rate was calculated by the percentage of apoptotic cells in the total cells. The experiment has been repeated for three times and statistically analyzed using the prism.

### Statistical analysis

The statistical analysis was performed by the Prism v.8.0.2 software (GraphPad). Statistical significance was also determined using the prism. A *p* value < 0.05 was statistically significant (*p < 0.05; **p < 0.01; ***p < 0.001; and ****p < 0.0001).

## Supplementary Information

Below is the link to the electronic supplementary material.

Supplementary file1 (PPTX 2776 KB)

## Data Availability

All data generated or analyzed during this study are included in this published article.
