# Supplementary material for: Cuproptosis-related gene SLC31A1 expression correlates with the prognosis and tumor immune microenvironment in glioma
Source: Funct Integr Genomics. 2023 Aug 23;23(3):279. doi: 10.1007/s10142-023-01210-0 (PMC10447603; doi:10.1007/s10142-023-01210-0)
Supplement: Supplementary file 1 — Supplementary file1 (PPTX 2776 KB) [file 10142_2023_1210_MOESM1_ESM.pptx]

## Slide 1
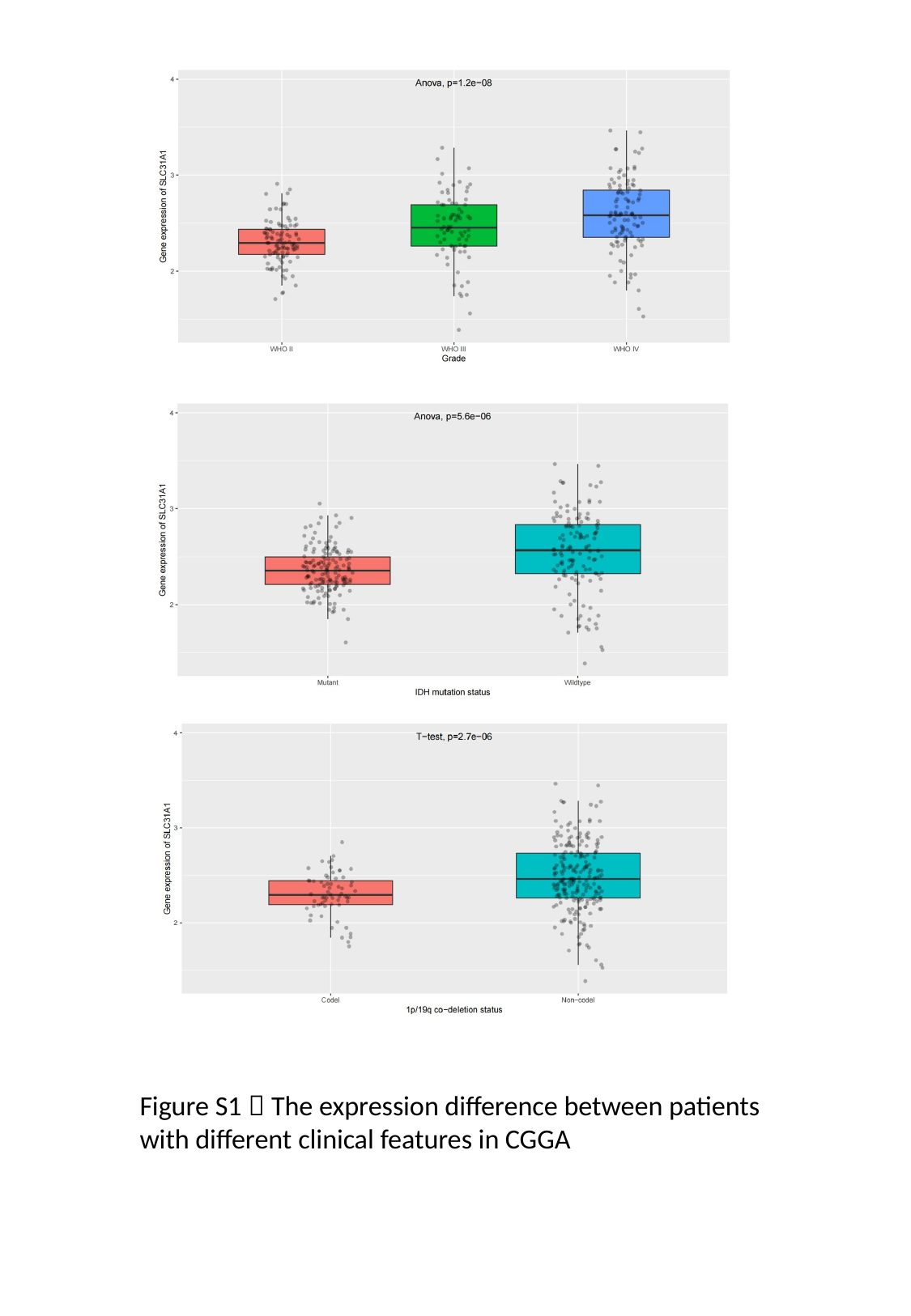

Figure S1：The expression difference between patients with different clinical features in CGGA

## Slide 2
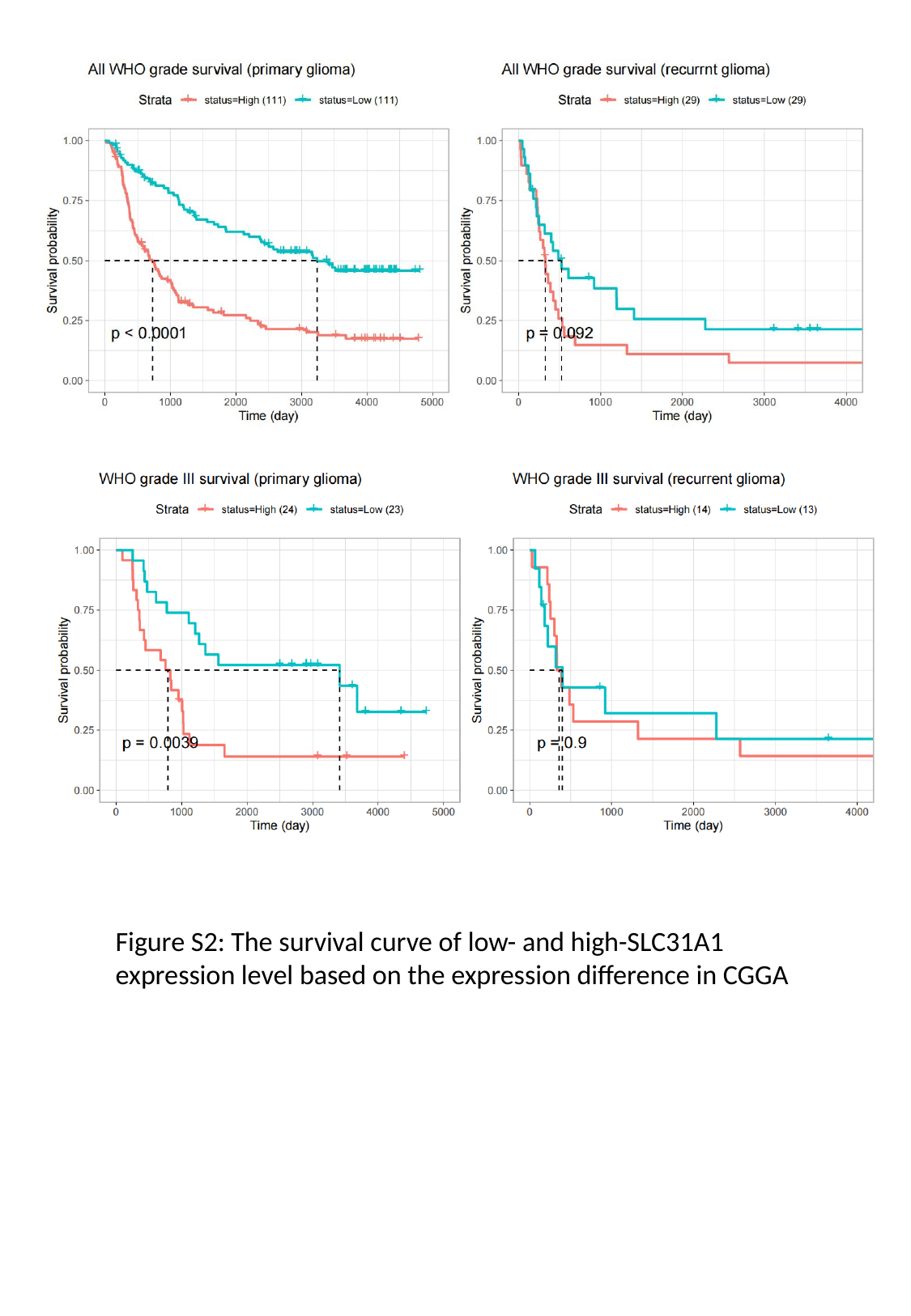

Figure S2: The survival curve of low- and high-SLC31A1 expression level based on the expression difference in CGGA

## Slide 3
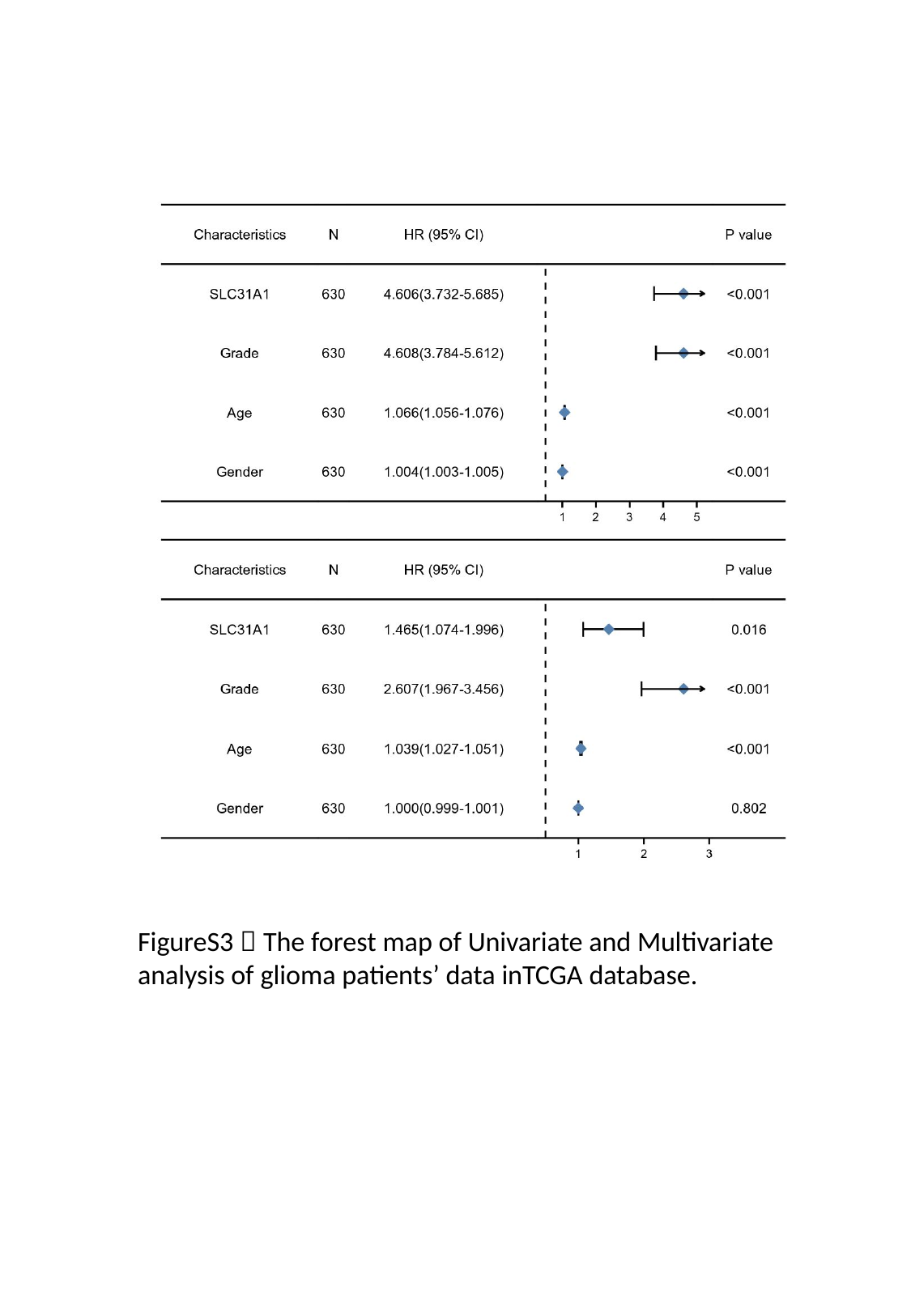

FigureS3：The forest map of Univariate and Multivariate analysis of glioma patients’ data inTCGA database.

## Slide 4
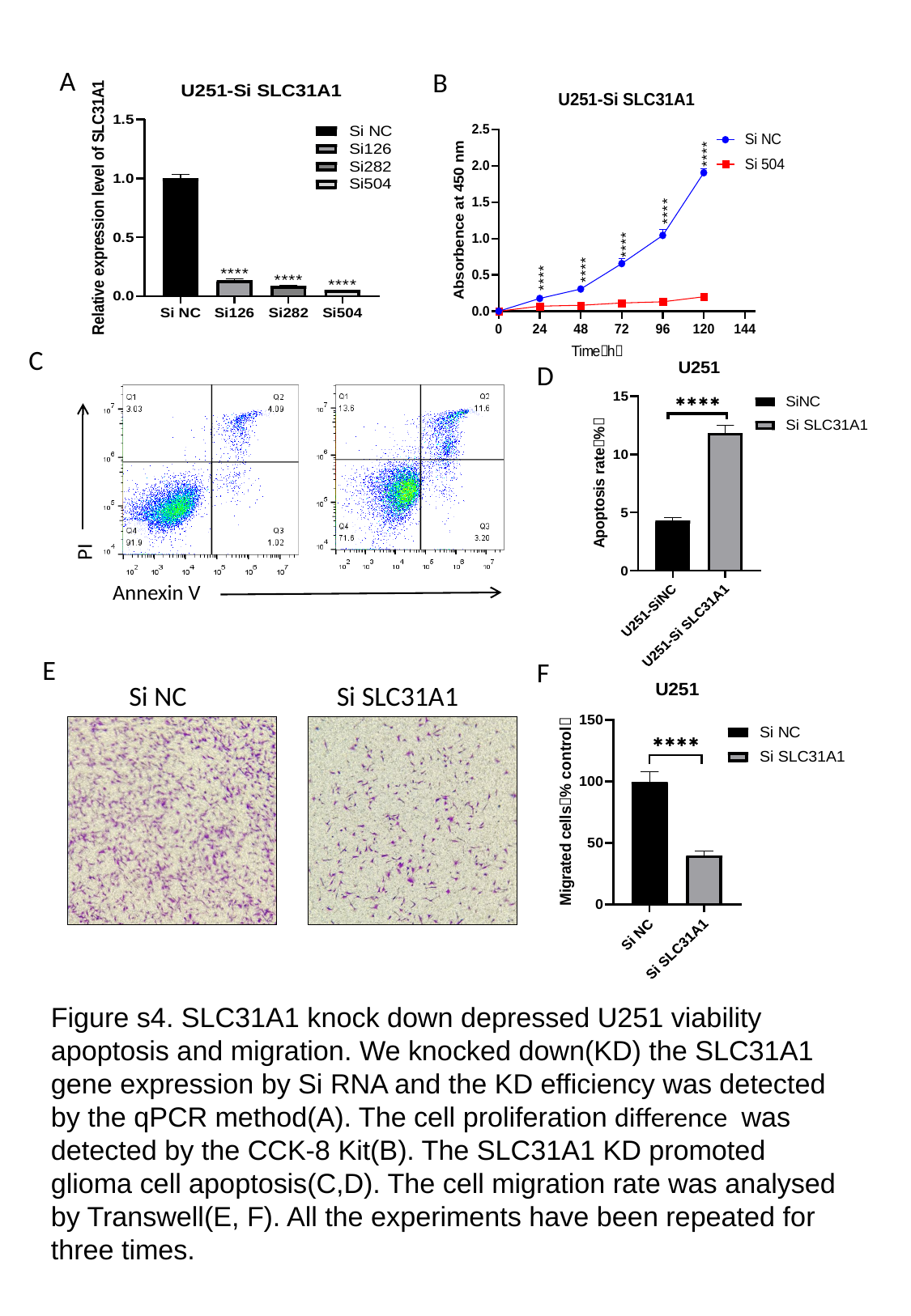

A
B
C
D
PI
Annexin V
E
F
Si NC
Si SLC31A1
Figure s4. SLC31A1 knock down depressed U251 viability apoptosis and migration. We knocked down(KD) the SLC31A1 gene expression by Si RNA and the KD efficiency was detected by the qPCR method(A). The cell proliferation difference was detected by the CCK-8 Kit(B). The SLC31A1 KD promoted glioma cell apoptosis(C,D). The cell migration rate was analysed by Transwell(E, F). All the experiments have been repeated for three times.
